# Primary Spinal Infections in Patients With Hematologic Immunocompromising Conditions: A Systematic Literature Review

**DOI:** 10.5435/JAAOSGlobal-D-22-00178

**Published:** 2023-05-08

**Authors:** Naomie Devico Marciano, Ryan S. Beyer, Andrew Nguyen, Anushka Paladugu, Matthew H. Hatter, Austin Franklin, Nolan J. Brown, Gaston Camino Willhuber, Nitin Bhatia, Michael Y. Oh, Yu-Po Lee

**Affiliations:** From the University of California, Irvine School of Medicine, Irvine, CL, (Dr. Marciano and Ms. Paladugu); the Department of Orthopaedic Surgery, University of California, Irvine, CL, (Mr. Beyer, Mr. Hatter, Mr. Franklin, Dr. Willhuber, Dr. Bhatia, and Dr. Lee); and the Department of Neurological Surgery, University of California, Irvine, CL, (Mr. Nguyen, Mr. Brown, and Dr. Oh).

## Abstract

Primary spinal infections (PSIs) are a group of infectious diseases characterized by inflammation of the end plate-disk unit or its surroundings. PSI is considered more prevalent and aggressive among patients with chronic immunocompromised states. Association of PSIs, immunocompromising cancers, and hemoglobinopathies has not been systematically analyzed. We conducted a systematic review to study characteristics, clinical presentation, and mortality of patients with PSI in the setting of hematologic disease.

**Methods:**

A systematic literature search in PubMed, Web of Science, and Scopus was conducted in April 2022 in accordance with Preferred Reporting Items for Systematic Reviews and Meta-Analyses guidelines. We included retrospective case series and individual case reports.

**Results:**

On careful review, 28 articles published between 1970 and 2022 were selected. These studies featured 29 patients who met inclusion criteria (mean age 29 years, age range 1.5 to 67 years; 63.3% male). Lumbar infection was the most common location (65.5%), with *Salmonella* (24.1%) as the main causative microorganism. Neurologic compromise was present in 41% of patients, and surgical intervention occurred in 48.3%. Average antibiotic duration was 13 weeks. The postoperative complication rate was 21.4%, with a mortality of 6.9%.

**Conclusion:**

PSI in patients with hematologic disease, while having shorter periods to diagnosis, presents increased rates of neurologic deficit, surgical intervention, and complications.

Primary spinal infections (PSIs) include conditions such as discitis which involves the intervertebral disk, spondylodiscitis which involves the intervertebral disk and end plate of the vertebral body, and vertebral osteomyelitis which involves infection and inflammation of the vertebra. PSIs have been characterized in the setting of comorbidities that leave patients especially susceptible to fungal, viral, and bacterial vertebral infections. These comorbidities include solid organ transplant patients, patients with chronic conditions such as diabetes mellitus and liver cirrhosis, and patients with autoimmune conditions. It is understood that PSIs are more prevalent and aggressive among patients in chronic immunocompromised (IC) states. Thus, the authors sought to investigate PSIs in the context of hematologic diseases, particularly those that affect the immune system.

Owing to the nonspecific symptoms such as fever and generalized back pain, an undiagnosed, rapidly developing PSI can pose a serious threat. Patients often display better outcomes when PSIs are diagnosed earlier in the course of the infection. Therefore, diagnosis and rapid intervention to treat PSIs is of paramount importance. Interventions include intravenous or oral antibiotics and more invasive surgical methods, such as abscess drainage or decompressive laminectomy for extensive infections.^1-3^

In this review, we focused on PSI patients with hematologic diseases, which included specifically sickle cell disease (SCD), acute lymphoblastic leukemia (ALL), acute myeloid leukemia (AML), multiple myeloma (MM), Hodgkin lymphoma (HL), non-Hodgkin lymphoma (NHL), alpha thalassemia, beta thalassemia, and hemolytic anemia. In each of these hematologic diseases, immunosuppression is often the result of both the disease and the regimens that are used to treat these conditions. Although it is commonly accepted that patients suffering from hematologic diseases are at an increased risk of infection, these have not been studied in the context of PSI.

The etiologies of infection vary depending on the type of immune dysfunction. Usually, patients with malignancies, such as ALL and chemotherapy-induced neutropenia, present with infections of fungal origin. As demonstrated by investigations into PSI and IC solid organ transplant recipients, invasive fungal infections are aggressive and associated with high mortality rates.^4,5^ The treatment of ALL commonly includes a combination of chemotherapy, radiation, and stem cell transplant—a regimen often accompanied with a neutropenic phase of increased susceptibility to infection.^6^ Although ALL is the most common leukemia in children, AML is the most common leukemia in the adult population. In this population, infections represent 3% to 7.3% of deaths during chemotherapy treatments.^7^ In MM, both cellular and humoral-mediated immunities may be impaired, leading to a notable increase in morbidity and mortality caused by infection.^8^ Infections are most common during the active period of the disease while immunosuppression is the cumulative result of the disease itself and the treatments patients with MM receive.^9^ In MM, the infections are most often caused by encapsulated bacteria. Hodgkin lymphoma and non-Hodgkin lymphoma are both cancers characterized by abnormal lymphocyte proliferation, which might result in a weakened immune response. In addition, the treatment of these lymphomas includes chemotherapies, which decrease the ability of the immune system to fight infections.^10^ Additional hematologic diseases such as alpha thalassemia and beta thalassemia, characterized by an absence or reduction in the alpha globin or beta globin chain of hemoglobin, respectively, result in hemolysis and impairment of erythropoiesis. In beta thalassemia, infection constitutes the second most common cause of mortality.^11^ In this condition, the increased risk of infection is attributed to both the recurrent need for transfusions and a coexisting immune deficiency.^11^ Other hemoglobinopathies, such as SCD and hemolytic anemia, are also correlated with a deficient immune response. In patients with SCD, the spleen is the first organ to be affected.^12^ Splenic damage resulting from SCD reduces IgM levels, which increases the risk of encapsulated bacterial infections.^13^ Furthermore, children with SCD are predisposed to osteomyelitis as increased hematopoiesis compensates for chronic hemolysis.^13^ In the case of SCD, functional hyposplenia or asplenia caused by the sickle cells results in a decreased ability to filter phagocytic cells and produce antibodies.^14^ Morbidity and mortality in SCD are commonly caused by encapsulated bacteria *Salmonella spp*. and plasmodium falciparum.^15^

To the best of our knowledge, no comprehensive systematic review concerning the overall incidence, characteristics, prognosis, and estimated mortality in patients with spinal infection secondary to hematologic disease has been conducted. Therefore, the objective of this study was to analyze—through a thorough systematic literature review—the baseline characteristics, clinical presentation, and mortality of patients with spinal infection in the setting of hematologic disease.

## Methods

### Eligibility Criteria

We included retrospective case series and singular case reports analyzing the number of patients, baseline characteristics, clinical presentation, causative microorganism, treatment, duration of antimicrobial treatment, postoperative complications, follow-up duration, and mortality. We excluded studies conducted in animals and cadavers. We only included studies written in English because the reviewers could only ensure accurate analysis in this language. We did not define any other exclusion criteria. All other reported cases in which patients with hematologic disease (SCD, beta thalassemia myeloma, lymphoma, leukemia) eventually developed spinal infection were included.

### Information Sources

A systematic literature search was conducted in PubMed, Web of Science, and Scopus databases in April 2022, to identify studies reporting the outcome of spinal infection in patients with hematologic disease. The search strategy was developed by one reviewer (A.N.) by consulting the Peer Review Electronic Search Strategies criteria.^16^ The search strategy for PubMed, Web of Science, and Scopus was as follows: leukaemia OR lymphoma OR myeloma OR sickle OR sickle AND cell OR anemia OR hemophilia OR thalassemia OR hbc OR hemoglobin AND spondylodiscitis OR discitis OR vertebral osteomyelitis. Study selection and data extraction were done by two reviewers (A.N, R.B), and data entry was checked by one reviewer (G.C.W.). When disagreements arose, consensus was achieved after consulting a third reviewer (G.C.W.). Full articles were retrieved when reviewers found titles and abstracts potentially relevant based on the inclusion criteria, i.e. included spondylodiscitis patients with infections secondary to hematologic disease. The data from each study were then extracted and analyzed for age, sex, time from onset of local pain to diagnosis, region of spine infection (cervical, thoracic, lumbar), causative organism, inflammatory markers, comorbidities, neurologic deficit outcome, duration of antimicrobial treatment, surgical outcome morbidity, and mortality. If a study did not explicitly mention the presence of a clinical characteristic, it was assumed the characteristic was not present in that patient set.

### Selection of Sources of Evidence

### Data Charting Process and Data Items

Data were collected in a table by two reviewers (A.N., R.B.) while another reviewer (G.C.W.) monitored all entries for completeness and accuracy. We collected the following data from the included studies: author, publication year, study design, number of patients, patient characteristics, treatment, antibiotic duration, and follow-up length.

### Critical Appraisal of Sources of Evidence

Being that this is a systematic review, no risk of bias assessment existed.

### Synthesis of Results

We provide a descriptive and analytical review of the studies included in this review along with a narrative and tabular summary of the data collected. We analyzed similarities and differences within and in between studies to identify patterns and offer an explanation for the findings. Furthermore, we developed an evidence map illustrating the baseline characteristics, clinical presentations, and outcomes for hematologic disease patients diagnosed with spinal infection. This systematic review was reported in accordance with the Preferred Reporting Items for Systematic Reviews and Meta-Analyses.^16^

## Results

### Selection of Sources of Evidence

**Figure 1:**
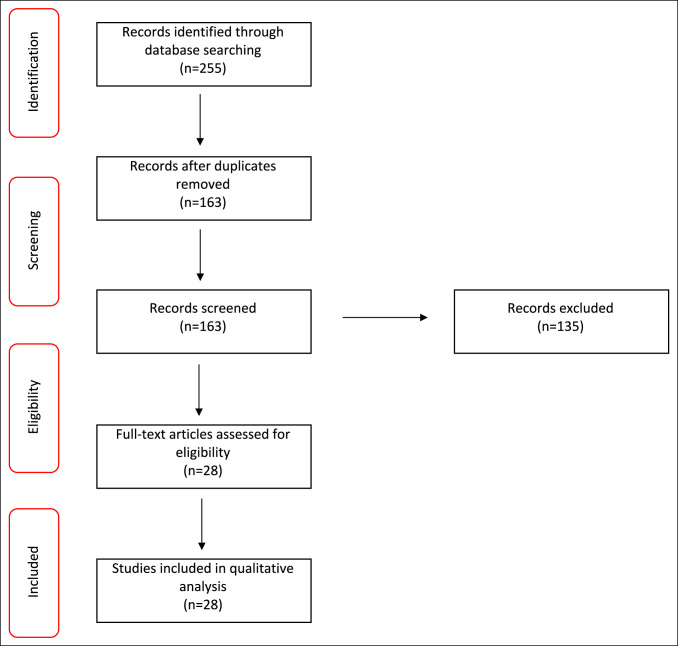
Preferred Reporting Items for Systematic Reviews and Meta-Analyses study selection flow diagram.

Figure 1 shows the study selection process. The literature search initially identified 255 studies (94 from PubMed, 94 from Web of Science, and 67 from Scopus). Reference sections were scrutinized to ensure that relevant studies were not missed, yielding no additional studies, and duplicates (56 duplicates; 36 triplicates; 92 total) were removed. In total, 28 articles published between 1970 and 2022 were selected based on the inclusion and exclusion criteria for the present systematic review. These included 1 case series and 27 case reports (Table 1).

**Table 1:** Demographic and Clinical Variables of Included Studies

Authors & Year	Study Design	No. of Pts	Age (yrs), Sex	Hematologic Condition	Spinal Pathology	Pain	Neurologic Deficit	Main Microorganism	Surgery	Antimicrobial Treatment Duration	Follow-up
Kooy et al., 1996^25^	CS	2	Mean 32; 1M, 1F	SCD (2)	T12-L1 spondylodiscitis (1)	2	0	*Staphylococcus* (2)	n/a	Mean 7 wk	n/a
Al-Tawfiq, 2008^26^	CR	1	18, F	SCD	L3-L4 VO	1	0	*Bacteroides fragiles*	n/a	6 wk	n/a
Krupniewski et al., 2012^27^	CR	1	39, F	SCD	T9-10 spondylodiscitis	1	0	*Mycobacterium tuberculosis*	n/a	n/a	n/a
Gardner, 1985^28^	CR	1	2, M	SCD	L3 VO	0	0	*S. enteriditis*	L3 laminectomy	4 wk	n/a
Li et al., 2016^29^	CR	1	17, F	Hemolytic anemia	L1 VO	1	1	*Cryptococcus neoformans*	Thoracolumbar débridement with posterior fusion	12 wk	3 mo
Morgan et al., 2006^30^	CR	1	20, M	SCD	Lumbar VO	1	0	*Salmonella*	n/a	6 wk	1.5 mo
Martino et al., 1990^31^	CR	1	15, M	SCD	T5-T8 VO	1	1	*S. enteriditis*	Thoracic laminectomy and T7-T8 left costotransversectomy	6 wk	36 mo
Elnour et al., 2022^32^	CR	1	27, M	SCD	0T5-6 spondylodiscitis	1	0	*Salmonella*	T4-T6 laminectomy	4 wk	n/a
Asnani et al., 201033	CR	1	30, M	SCD	L2-L3 VO	1	0	*Bacteroides*	n/a	8 wk	12 mo
Yazici et al., 2005^34^	CR	1	13, F	NHL	L3-4 spondylodiscitis	1	1	n/a	n/a	6 wk	15 mo
Farrar et al., 2015^35^	CR	1	60, M	α-Thalassemia	L5-S1 discitis	1	1	*S. enterica*	L3-L4 laminectomy	8 wk	n/a
Griguolo et al., 2015^36^	CR	1	30, M	HL	S1-S2 VO	1	0	n/a	n/a	8 wk	20 mo
Koehler et al., 2018^37^	CR	1	56, M	HIV-associated Burkitt lymphoma	L3-L4 spondylodiscitis	1	0	*C. albicans*	Vertebral bone resection and dorsal/ventral spine fusion	24 wk	n/a
Antonio et al., 1994^38^	CR	1	14, F	ALL	L3-4 discitis	1	1	*B. capitatus*	L4 débridement	n/a	12 mo
Weiss et al., 1970^39^	CR	1	37, M	SCD	C7-T1 VO	1	0	*S. utah*	n/a	4 wk	12 mo
Devrim et al., 2005^40^	CR	1	5, M	SCD	T10-T11 VO	1	0	*S. paratyphi* B	Anterior vertebrectomy and posterior instrumentation and fusion	2 wk	12 mo
Makay et al., 2009^41^	CR	1	6, M	AML	VO	1	1	n/a	n/a	None	12 mo
Beluffi et al., 2008^42^	CR	1	1.5, M	AML	T1-T2 VO	0	1	*Aspergillus*	Débridement	28 wk	n/a
McCaslin et al., 2015^43^	CR	1	19, F	ALL	T12-L1 spondylodiscitis	0	1	*Aspergillosis*	T11-L1 laminectomy	3 wk	n/a
Mikic et al., 2014^44^	CR	1	43, F	HL	L2-L3 spondylodiscitis	0	1	*R. equi*	n/a	56 wk	96 mo
Burton et al., 1998^45^	CR	1	47, M	MM	L2-L3 discitis	1	0	*S. aureus* and *Mycobacterium tuberculosis*	n/a	20 wk	12 mo
Camacho et al., 2002^46^	CR	1	67, M	MM	L1-L2 discitis	1	0	*S. lugendenis*	n/a	56 wk	36 mo
Cevolani et al., 2021^47^	CR	1	57, M	AML	L3-L5 spondylodiscitis	1	1	*C. Tropicalis*	n/a	n/a	12 mo
Fung et al., 2018^48^	CR	1	16, M	ALL	T10-T12 VO	1	1	*Rhizopus*	T11-T12 débridement, thoracotomy with T10–12 dissection	n/a	24 mo
Lott-Duarte et al., 2008^49^	CR	1	6, F	SCD	Spondylodiscitis	1	0	n/a	n/a	24 wk	n/a
Park et al., 2000^50^	CR	1	37, M	ALL	L3-S1 discitis	1	0	*Aspergillus tereus*	Débridement and fusion	n/a	n/a
Supreeth et al., 2020^51^	CR	1	50, M	SCD	L4-L5 spondylodiscitis	1	0	*C. aureus*	L4-L5 decompression, débridement, and pedicle screw instrumentation	8 wk	6 mo
Weber et al., 2009^52^	CR	1	39, F	SCT	T8-T9 spondylodiscitis	0	1	*Mycobacterium tuberculosis*	T8-T9 laminectomy	n/a	18 mo

ALL = acute lymphoblastic leukemia, AML = acute myeloid leukemia, CR = case report, CS = case series, HL = Hodgkin lymphoma, MM = multiple myeloma, NHL = non-Hodgkin lymphoma, SCD = sickle cell disease, SCT = sickle cell trait, VO = vertebral osteomyelitis

### Synthesis of Results

The sample size across all studies was 29 patients, with all remaining after application of the inclusion and exclusion criteria. This cohort of 29 patients exhibited an age range of 1.5 to 67 years. The median age was 30 years. There were 19 male patients (63.3%). Etiologies for spinal disease were reported for all 29 patients, which included 44.8% vertebral osteomyelitis (n = 13), 37.9% spondylodiscitis (n = 11), and 17.2% discitis (n = 5).

### Clinical Presentation

Of the 29 patients included in this synthesis, 13 (44.8%) presented with SCD, 4 (13.8%) presented with ALL, 3 (10.3%) presented AML, 2 (6.9%) presented with MM, 2 (6.9%) presented with HL, 2 (6.9%) presented with NHL, 2 (6.9%) presented with beta thalassemia, 1 (3.4%) presented with a sickle cell trait, 1 (3.4%) presented with alpha-thalassemia, and 1 (3.4%) presented with hemolytic anemia. Of the 29 patients, 82.8% (n = 24) presented with local pain, 75.9% (n = 22) with fever, 41.3% (n = 12) with neurologic deficits, and 27.6% (n = 8) with septic manifestation. 48.3% (n = 14) of patients presented with an elevated erythrocyte sedimentation rate. 44.8% (n = 13) of patients presented with elevated C-reactive protein. 34.4% (n = 10) presented with an elevated white blood cell (WBC) count. 55.2% (n = 16) of patients presented with low hemoglobin levels. The level of spinal infection was reported for all 29 patients, of whom 3.4% (n = 1) presented with cervical infection, 44.8% (n = 13) presented with thoracic infection, 65.5% (n = 19) presented with lumbar infection, and 10.3% (n = 3) presented with sacral infection. The presence of epidural abscess was reported in 20.7% (n = 6) of patients. Other abscesses were reported in 34.5% (n = 10) of patients, and of those, included 60% psoas abscesses (n = 6), 20% paravertebral abscesses (n = 2), 10% paraspinal abscess (n = 1), and 10% unknown abscess (n = 1).

### Causative Microorganisms and Antimicrobial Regimen

Genus *Salmonella* was reported as the main causative microorganism in 24.1% (n = 7) of patients. Other causative microorganisms included genuses *Staphylococcus* 13.8% (n = 4), *Candida* 10.3% (n = 3), *Mycobacterium* (tuberculosis) 10.3% (n = 3), *Aspergillus* 10.3% (n = 3), *Bacteroides* 6.9% (n = 2), *Cryptococcus* 3.4% (n = 1), *R. equi* 3.4% (n = 1), *Rhizopus* 3.4% (n = 1), and *Blastoschizomyces capitatus* 3.4% (n = 1). Gram-negative microorganisms were found in 31% (n = 9) of patients. No causative microorganism was found for 13.8% (n = 4) of patients. Antimicrobial duration after medical/surgical treatment was reported for 22 patients, for whom the average duration of antimicrobial treatment was 13.2 weeks. No reports of antimicrobial treatment failure were noted.

### Methods of Surgical Intervention

The median time from presentation of symptoms to diagnosis of spinal infection was 3 weeks. The median time from presentation of symptoms to surgical intervention for spinal infection was 4.5 weeks. In total, 48.3% (n = 14) of patients underwent surgical intervention. A decompressive laminectomy alone was reported for 29.6% (n = 5) of surgical patients, débridement alone for 14.3% (n = 2), débridement and fusion for 14.3% (n = 2), vertebrectomy and fusion for 14.3% (n = 2), débridement and laminectomy for 7.1% (n = 1), débridement and spondylectomy for 7.1% (n = 1), and débridement and laminectomy and instrumentation for 7.1% (n = 1).

### Postoperative Complications and Mortality

Postoperative complications occurred in 21.4% (n = 3) of the 14 patients who had surgery. One of these patients with postoperative complications had a T2-T3 and C7 débridement and died a few days after, of which CT revealed hydrocephalus and transependymal CSF leakage. This patient had a history of AML, for which they were being treated with hematopoietic stem cell treatment. Another patient with postoperative complications had a T12-L1 laminectomy and likewise, died soon after the operation. The infection spread to the cerebral regions leading to complications including cerebral infarction. Aspergillus was the primary causative microorganism for both patients who ultimately died. The final patient with postoperative complications had a T7-T9 laminectomy with T7-T11 instrumentation. Imaging and secondary surgery were ordered after ensuing neurologic deficits, of which IV heparin was administered to address detected ischemia of a spinal artery. Follow-up duration was reported for 17 patients, for whom the average follow-up duration was 19 months. In total, two patients (6.9%) from our study died with a 30-day mortality of 6.9%.

## Discussion

In recent years, the advancement in treatments for hematologic diseases has caused an increase in the size of the IC patient population. This has led to a concomitant increase in morbidity and mortality in part due to fungal and bacterial infections. PSI in IC patients might behave differently in epidemiology, clinical presentation, and outcomes compared with nonimmunocompromised patients.

Patients affected by hematologic diseases are at increased risk of infection-related morbidity and mortality because of their IC state, secondary to both the primary disease and treatments they receive. The lack of symptom specificity in PSI requires an expansive workup and high level of suspicion; this could be even more difficult in IC patients. Primary symptom identification relies on the presence of localized pain observed in 82.7% of our patients, fever noted in 75.8%, and neurologic deficits reported in 41.3%. The prevailing benchmark diagnosis for PSI comprises both identification of the pathogen and diagnostic imaging, specifically MRI.^17^ Pathogenic identification may require an open biopsy if blood and tissue cultures are negative.^17^ Prompt identification of the invading microorganism is critical in reducing risk of complications secondary to PSI, ranging from neurologic deficits to persistent deep tissue infections with progressive deformity, and chronic pain or death.

The current standard of care maintains conservative measures to identify and treat the causative organism in patients with PSI. For example, in PSI complicated by an epidural collection, compression of the spinal cord or nerve roots, spinal instability and deformity, as well as progressive or acute neurologic deficits, surgery has been identified as the standard of care. In our cohort of 29 patients, 14 (48.3%) underwent surgery, specifically decompressive laminectomy (29.6%), débridement alone (14.3%), débridement with fusion (14.3%), and vertebrectomy with fusion (14.3%). Of the 14 patients who underwent surgery, 8 (57.1%) displaced neurologic compromise. Notably, in our cohort, 41.3% of patients (12 of 29 patients) demonstrated some neurologic compromise, which is higher than the 23% reported in nonimmunocompromised patients by Pola et al.^18^

Patients with hematologic disease are IC and thus are at higher risk of contracting more numerous and aggressive infections. Close monitoring of their condition by their primary oncologists may explain why the diagnosis of PSI in this population was on average faster (29.3 days) compared with the 2 to 6 months’ delay observed by Waheed et al or with the 49.9 days’ delay observed by Pola et al.^18*,*19^ It is important to highlight that the lag time in diagnosis is correlated with higher rates of mortality and complications.^1-3^ However, when considering that the extent of infection can be used as a proxy for severity of symptoms and the need for surgical intervention, the immunosuppressed state of this patient population may contribute to the higher number of patients who underwent surgery. In our cohort, 48.3% patients required surgery (14 of 29 patients) while Pola et al^20^ reported that 22.75% (47 of 207 patients) of their cohort required surgical treatment. Furthermore, in a different study, Pola et al. reported that 60.5% of their patients were treated conservatively.^21^ It is, therefore, possible to hypothesize that the higher rate of surgery needed in the IC population is because of infections spreading faster and resulting in more aggressive complications. Moreover, in this cohort, 21% of patients who underwent surgery experienced complications, which is higher than the 8.5% reported in the general PSI patient population by Pola et al.^20^ Therefore, although in the IC population, the diagnosis of PSI is made earlier, an important factor in better overall outcome is that prompt diagnosis does not translate to lower rates of complications in the IC population.

Notably, the infections observed in this immunologically compromised cohort varies from the expected causative microorganisms in other patients with spondylodiscitis. It has been reported that in spondylodiscitis, the most common microorganisms involved are *Mycobacterium Tuberculosis* and *Staphylococcus* species; however, in our study, infections with *Salmonella spp* (24.1%) were more common, followed by *Staphylococcus* (13.8%).^21,22^ This finding highlights the fact that in patients with underlying hematologic immunocompromising conditions, PSI often results from less expected causes. Notably, in this cohort, PSI often develops from an infection with the most expected microorganism for that particular disease. This is the case for the four patients with ALL, who all developed PSI caused by fungi, which is the most common infection risk in patients with persistent neutropenia induced by chemotherapy treatments.^23,24^ Furthermore, in this cohort, 44.8% of patients had SCD, of which 53.8% (7 of 13) developed PSI from *Salmonella spp* infection, an expected source of infection in patients with SCD.^8^ Interestingly, the two patients who died in our review both were infected with *Aspergillus* species, one with AML and other with ALL. In a recent systematic review of PSIs in solid organ transplant patients currently in journal submission, the aggressive nature of invasive fungal infections yielded a relatively higher rate of postoperative mortality. This parallels the trend observed in our review of spinal infections in patients with hematologic disease and emphasizes the importance of early identification and treatment implementation. In summation, owing to the importance of rapid diagnosis and treatment on the better outcome of patients with PSI, it is crucial to consider this differential in IC patients presenting with any new back pain even when the patients present with less usual infections of this condition.

### Limitations

Our study has some limitations. First, all data are provided by case series and case reports; therefore, the quality of this study relies on the quality of the included articles. However, considering the relatively rare occurrence of this condition, this is the best and most recent evidence related to spinal infection in patients with hematologic disease. Moreover, most of the data from patients with PSIs comes from case series and case reports, with a low number of higher level of evidence studies in this field. In the future, it may be useful to explore similar questions with group designation in those with and without hematologic ailments, by case-control assignments. This study brings up new questions again as to how differently clinical progression ensues in various dynamics of comorbidities; controlled, intrastudy comparisons would allow for more robust implications to draw upon. Despite these limitations, this is the first systematic review to our knowledge regarding PSI in this population, overall providing a novel value.

## Conclusion

This study adds to the current body of PSI in the context of hematologic disorders. Notably, our results suggest higher rates of neurologic deficits, surgical intervention, and associated complications despite prompter diagnosis times in IC populations. Other distinctions include infective causes most consistent with the immunologic disorder rather than the current pathology, in this case, PSI. These trends are worth noting when tailoring PSI treatment plans for individuals with hemoglobinopathies, among other comorbidities. Nonetheless, the heterogenic clinical presentation is seldom limited to this population alone. The variation present in this study,^25,26,27,28,29,30,31,32,33,34,35,36,37,38,39,40,41,42,43,44,45,46,47,48,49,50,51,52^ consistent with overall spinal infection cases, urges additional research within other subsets to construct reliable diagnosis patterns. Because this is the first systematic review of PSI in a previously unexplored population, its purpose is to supplement the aforementioned goal of ultimately guiding clinical decision making for PSI patient populations.
